# Understanding the needs and challenges of unpaid carers caring for someone with drug and alcohol dependency: findings from a national qualitative evaluation

**DOI:** 10.1080/17482631.2025.2500395

**Published:** 2025-05-13

**Authors:** Sarah Tickle, Sarah Greenhow

**Affiliations:** Department of Criminology, Liverpool John Moores University, Liverpool, UK

**Keywords:** Carers, drug, alcohol, dependency, mental health, social harm

## Abstract

**Purpose:**

The cost-of-living crisis witnessed in the UK, in addition to the repercussions of the COVID-19 pandemic, has exacerbated growing concerns about its disproportionate impact on caregivers. All caregivers face unprecedented challenges, but this is further intensified when caring for someone with drug and alcohol dependency which this paper argues are direct social harms.

**Methods:**

This paper presents findings from an external evaluation of three projects from a wider national programme funded by NHS England and Improvement [NHSE/I]. Identified by NHSE/I as a vulnerable community, interviews were conducted with eight caregivers who were caring for someone with drug and alcohol dependency, which this paper focuses on.

**Results:**

After thematic analysis was conducted on qualitative data, key themes emerged that emphasized a range of specific challenges faced by unpaid carers, including stigmatization and marginalization, a lack of understanding about drug and alcohol dependency, and caring for complex needs, which were particularly challenging in accessing appropriate support.

**Conclusions:**

Caregivers overwhelmingly advocated for the urgent need to raise awareness around the role of being a “carer,” to see more individualized support provided, in addition to the recognition of treating mental health alongside drug and alcohol dependency in a supportive holistic approach.

## Introduction

The most recent estimate on the number of unpaid carers in the UK is around 10.6 million, meaning that 1 in 5 adults are providing care (Carers UK, 2022b). In this paper, when referring to unpaid carers and caregivers, the authors refer to people providing care which can be ‘anyone – a child or adult’ – who looks after a family member, partner or friend who needs help because of their illness, frailty, disability, a mental health problem or an addiction and cannot cope without their support. The care they give is unpaid’ (NHS England, 2021, p. 1). Within this context, there have been growing concerns in relation to the health and wellbeing of unpaid carers, but also the additional impact this has on those caring for someone from diverse and socially excluded populations.

Recognizing that caregivers face inequity because of their caring role initiated a clear commitment within The NHS Long Term Plan [LTP] (2019) to tackle health inequalities by identifying caregivers from vulnerable communities^1^ so that they would benefit from greater recognition and support. Since the publication of NHS (2019), it is evident that much has changed. Carer’s UK (2020a) reported that the COVID-19 pandemic had resulted in millions of new carers, with 4.5 million new to caring since the start of the COVID-19 pandemic. In addition, the cost-of-living crisis in the UK has raised further concerns about the impact of rising costs on unpaid carers in the future (Carers UK, 2022a). In 2023, 82% of caregivers reported that caring had a negative impact on their physical and mental health (Carers UK, 2023). Recognizing that caregivers experience worse health than non-caregivers led to Public Health England (2021) acknowledging that caring responsibilities should be considered a social determinant of health. In response to growing concerns that some unpaid carers do not feel confident accessing support from NHS services, NHS England and NHS Improvement [NHSE/I] launched Mind The Gap, a programme which sought to evaluate what works and what does not work when engaging with unpaid carers from vulnerable communities. The aim of the programme was to understand the barriers to accessing support services that caregivers from diverse and socially excluded populations experienced. NHSE/I funded a set of projects at a number of local sites across England in order to understand these barriers and to develop a set of recommendations created by the perspectives of unpaid carers themselves. NHSE/I identified two vulnerable communities as the Armed Forces and Veterans community (Greenhow & Tickle, in press); and drug and alcohol dependence, as areas with a particular lack of evidence base. Three projects supporting carers who were caring for someone with drug and alcohol dependency were selected for external evaluation by NHSE/I, which this paper focuses on. Therefore, given the importance and value of unpaid caring, this paper has national significance, and will also have relevance to international audiences, in relation to understanding the experiences of unpaid carers caring for someone with drug and alcohol dependency.

## Caring for someone with drug and alcohol dependence

Understanding the complex needs and challenges that people face when caring for someone with drug and alcohol dependence was an area of vital importance to NHSE/I for evaluation. There is still a lack of data related to the impact of caring for someone with drug and alcohol dependency, despite alcohol misuse being the biggest risk factor attributable to early mortality, ill health, and disability in England (Public Health England, 2016, p. 14). In addition, the number of deaths by drug poisoning has increased significantly and is the highest since records began in 1993 (Office for National Statistics, 2024). From this, it is evident that alcohol and drug misuse is highly associated with significant health and social problems (see Barber & Crisp, 1995; Chartier & Caetano, 2010) for the person with dependency, as well as impacting on the relationships with others (Public Health England, 2016) causing emotional distress and impacting on mental health and wellbeing (Casswell et al., 2011; cited in Public Health England, 2016, p. 15).

It has been well documented that relatives and close family members of people with drug and alcohol problems suffer “stress-related physical and psychological symptoms that can be severe and long-lasting” (see Copello et al., 2000, p. 331; Orford et al., 1998) and as a result, make more use of medical services (Roberts & Brent, 1982; Svenson et al., 1995). In relation to this, drug and alcohol-related harms associated with health disorders and disease, poor quality of life, financial struggles, impact on family relationships and friendships apply to both the caregiver and the person being cared for.

When exploring drug and alcohol dependency, a prevalent problem that exacerbates and hinders the issue is the way in which the media documents and reports this, often misrepresenting and “demonizing” drug use and its effects (UK Drug Policy Commission UKDPC, 2010, p. 6). As Taylor (2008), p. 371) suggests, there is “undoubtedly a dominant stereotypical image [of drug users] that has emerged, prevailed and been sustained within the reporting of national and local news over the last three decades”. This has been further explored by the Global Commission on Drug Policy, which reported in 2017 that perceptions of people who use drugs are often general assumptions that drug users are people living on the margins of society. These widespread misconceptions, the misrepresentation in the media, alongside drug control policies, constitute a vicious cycle’ (Global Commission on Drug Policy GCDP, 2017, p. 8). This has ultimately led to problems with treatment, intervention, and services, which have subsequently impacted those caring for their loved ones. This paper adds to the limited evidence related to unpaid carer experiences in this community and presents findings from an external evaluation of three projects funded by NHS England and Improvement [NHSE/I], which sought to understand the needs of unpaid carers in vulnerable and often marginalized communities.

## Methodology

The participants included in this paper are taken from an overall national external evaluation of Mind The Gap projects funded by the NHSE/I. There were 17 mind The Gap projects in which 448 carers participated and 10 projects were selected for external evaluation. Three projects supporting carers who were caring for someone with drug and alcohol dependency were selected for external evaluation by NHSE/I, which this paper focuses on. All projects were identified and selected by NHSE/I through a bidding process and the authors were informed of the projects that had been successfully awarded the funding and became involved after the selection of the projects. Following consultation with NHSE/I representatives, four evaluation research questions were developed by the authors to reflect the aims of the Mind The Gap programme (see Appendix 1). From this, a semi-structured interview schedule was designed that formed the basis of our data collection with carers. The interview schedule (see Appendix 2) incorporated four areas which were (1) experience of being a caregiver; (2) barriers/challenges to accessing support; (3) hopes for the future; and (4) what can be learned from this programme.

Following the selection of the projects by NHSE/I, the authors worked closely with projects in order to proceed with the evaluation in a way that was culturally sensitive, did not impact any of the work already undertaken, and to ensure that each caregivers’ time was recognized in an appropriate way. In doing so, the authors provided equal numbers of online vouchers per participant. The authors took advice from project leads in relation to the most appropriate methods to use in each site with caregivers. The authors also took the needs and preferences of caregivers into account and made, it an option that they have someone with them when participating in the evaluation.

Throughout the evaluation period, there was a continuing climate of uncertainty due to the COVID-19 pandemic and the disproportionate impact that this was having on the communities that funded projects had been supporting and engaging with. Factors such as COVID-19 pandemic restrictions, including a lack of face-to-face engagement, made this a challenging time. Additional difficulties due to technology, digital exclusion and a caregivers time, among other struggles, were taken into consideration in the design of the data collection. Due to ongoing concerns relating to Covid-19, methods were adapted to ensure the safety of all involved. For example, the use of online and personal communication when face-to-face was not possible. Qualitative data was employed to collect in-depth views of unpaid carers to explore what was meaningful to them in understanding their caring role in a “conversation with a purpose” (Burgess, 1984, p. 102). The flexible qualitative approach allowed these methods to be carried out online by telephone, email, or face-to-face in acknowledging that interview strategies incorporate a range of research techniques (Noaks & Wincup, 2004, p. 78). This was to ensure accessibility for participants to engage with the authors and to use methods that were most comfortable and appropriate for each unpaid caregiver, which was at the forefront of our ethical concerns.

Whilst the methods of engagement were varied for each project, all participants were asked the same questions from the interview schedule (see Appendix 2) to ensure reliability and rigour and the authors did this across the evaluation with all 10 projects that were evaluated. The authors were commissioned by NHSE/I to consult with projects from the start in developing co-produced tools and measures with each project. Building upon successful relationships with projects prior to evaluation and through to the collection of data with unpaid carers, enabled the validation of findings through multiple sources and at multiple times throughout the study.

The voices and perspectives of unpaid caregivers were central to our external evaluation. Through the ethical design of our research, adhering to the ethical principles of the Declaration of Helsinki, ethical approval was gained by Liverpool John Moores University Research Ethics Committee Approval Reference: 21/LCP/007. Prior to data collection, all participants were informed of our role. It was deemed appropriate for project gatekeepers to identify and contact carers initially to see if they were willing to participate; to ensure a warm handover and to put in place any further support needed for their engagement with the authors, regarded as best practice (Noaks & Wincup, 2004). The authors provided all participants with a consent form and a participant information sheet outlining the aims of our evaluation, the voluntary nature of their participation, their right to withdraw at any time, and the protection of their data through anonymity and the use of pseudonyms. Written informed consent was gained from all participants. Confidentiality was of highest importance. Only a participant’s name and the project that they were involved were collected as personal data. Personal data was only accessible to the authors and was stored confidentially. The authors informed all participants that they would not be named in any of the authors’ publications. To ensure anonymity, the authors used pseudonyms in all transcripts and reports to protect the identity of individuals and organizations involved. The authors ensured confidentiality and anonymity of all participants at all times. A summary report was sent to all project teams and participants permission was gained to use the information provided in future publications. This also ensured approval and careful reflection of participants views were carefully and accurately reflected. Upon receipt of signed written informed consent data collection took place between February and May 2022.

## Data collection

All participants were asked the same questions by the authors about their caring role, the challenges and barriers that they faced, what mattered most to carers, and what their hopes for the future were. For project A,^2^ two interviews were conducted via video call on Microsoft Teams, and two interviews were conducted via telephone. The duration of the interviews ranged from 20 to 40 min. Our external evaluation of Project B^3^ involved two caregivers participating by answering the prescribed questions that the authors developed and sent to the project lead for the questions to be sent to each participant. Our external evaluation of Project C^4^ involved one interview via telephone, and one participant answered questions via email.

The 10 projects selected by NHSE/I aimed to explore and understand the needs of unpaid carers from a range of specific vulnerable communities defined by NHSE/I, including young adult carers, learning disabilities and/or Autism, LGBTQ+, Gypsy, Roma and Traveller, Armed Forces and Veterans; and Drug and Alcohol Dependence. Given the aims of the Mind The Gap programme, the authors conducted an external evaluation of these projects, in which 28 carers and 78 practitioners participated overall. One of the areas that NHSE/I commissioned the authors to explore was related to the needs of unpaid carers supporting someone with drug and alcohol dependence. This paper specifically focuses on the findings of three of those projects, A, B, and C, of which eight unpaid carers participated who were caring for someone with drug and alcohol dependency. The authors focused on three projects for the purposes of this paper, and the findings presented here are not representative of all unpaid carers who participated in all projects or are representative of the whole population of unpaid carers. Whilst recognizing that there were common themes across all of the caregivers the authors spoke to, this paper purposefully focuses on those caregivers who were caring for someone with drug and alcohol dependence. In addition, the qualitative design deliberately aimed to recruit a small sample of participants from each project to allow for in-depth discussions. This approach gathered valuable insights, but it must also be recognized that the participant accounts are not generalizable to the wider participants within each project or demographic location. This paper purposefully intended to give voice to unpaid carers who were caring for someone with alcohol and drug dependence within a particular location and time period. The deliberate motivation here is to give voice to and understand, the personal caregiving experiences of those caregivers, whose complex roles are often overlooked or not fully explored in the literature (Zarzycki et al., 2022, p. 569).

## Data analysis

Thematic analysis was performed on qualitative data to identify patterns across the data, including similarities and differences in experience and opinion across participants within and across sites. We carried out the thematic analysis following Braun and Clarke (2022) six phases involving: familiarizing yourself with the data, generating initial codes, searching for themes, reviewing themes, naming themes and producing the report. The process of developing themes was carried out through exploration of key themes related to each project and the evaluation research questions namely, experiences, barriers and challenges, hopes and recommendations and sharing key learning. Following the data analysis, a draft report listing all unpaid carers’ recommendations was prepared, in addition to the final report produced for NHSE/I/. This was shared with all unpaid carers who participated in the project to read and comment on. All caregivers provided feedback to shape the final co-produced version for each project to retain.

## Results

Five key themes were identified through the qualitative data collection.

1. The culture of blame, shame and stigma (Caregiver’s challenges)
2. Marginalization (Caregiver’s challenges)
3. Inclusion (Caregiver’s hopes for future)
4. Dual diagnosis (addiction and mental health) (Caregivers hope for future)
5. Mental Health and wellbeing (Caregiver’s hope for future)

## Findings

### Culture of blame, shame and stigma

Unpaid carers across the three projects were first asked about the challenges that they faced when caring for someone with drug or alcohol dependency. It was clear that their caring role was complex and unpaid carers faced several challenges, often experiencing feelings of shame and of “stigma” associated with drug and alcohol dependency. This was expressed by one unpaid carer, who claimed that “I suppose a lot of people will be quite ashamed of it, that their loved ones are misusing, or whatever. So, they don’t want to talk about the negatives in their lives” (Carer 7). It was evident that stigma and fear of being judged by others often resulted in unpaid caregivers feeling socially isolated. Isolation resulted from fear of feeling ashamed, as another carer expressed “I think isolation. I was ashamed. I did not want to meet people. I was a new mum as well” (Carer 2). The experience of feeling stigmatized was felt by another unpaid carer, “shame and stigma were a challenge for me—and meeting in a group of people going through the same worrying time really helped me. Also, I would think it would be harder now to find such a group” (Carer 1). This was reiterated by Carer 3, who clearly identified that one barrier to caring for someone was “a lack of support for carers and parents and the blame and that side of things, which nobody has identified before, nobody has wanted to listen” (Carer 3).

To overcome feelings of stigmatization in their caring role, caregivers expressed that they felt it was important that practitioners and the public were educated about the actual role of being caregivers so that assumptions were not made about the reality of caring:

For people to care, not just this charity but others, just to think and not judge, I never asked this for this to happen to me, in fact I don’t want to be a carer, but no one is going to do it for me. (Carer 6)

In addition to feeling judged unpaid carers also felt that support was difficult to access “it’s been a strange fourteen, fifteen years, my life is completely different, but I love him to bits but it’s a challenge, but he’s judged all the time and there is no support for the carers” (Carer 8). Feeling stigmatized by medical professionals and subsequently blamed for the situation was expressed by a parent caring for someone with drug and alcohol dependency:

My only thing is that somebody who maybe isn’t as strong as me needs more support in the system. My doctors were really supportive, but whenever I had to go with my son to, usually it was Accident and Emergency, when he’d done something or he was threatening to kill himself, or whatever I just didn’t feel as though I was being listened to. I felt that it was, Oh God, well, you are the mother of a druggie and an alcoholic. What do you expect? I just feel that maybe you don’t get quite the same treatment … You always think you are responsible, you have done something that has made them like that, you know. (Carer 1)

The impact of social and public stigma experienced by unpaid carers was again reported by another carer, where they felt blamed and discriminated against by professionals:

Over the years, it has been the blame culture of professionals, so being a parent, you are seen as a child’s mum, you are not seen as a person … But it’s when things are really bad and they’re taking attempted overdoses and you are still not getting the support and you are still reading the reports that are blaming you because you take them food parcels and things and they force you to make your child homeless before they will find a placement and you go from requesting urgent respite to being told you have been neglectful in writing and could be prosecuted for refusing. It’s not done to your face, it’s done in how things are written, it’s looking at reports and you read it, and the perceptions and knowing that it can be read in a different way. (Carer 2)

It became apparent that addiction was a stigmatized condition and that misconceptions about drug and alcohol dependency were emotionally difficult for one carer in their caring role:

A lot of people will be quite ashamed of it, that their loved ones are misusing, or whatever. So, they don’t want to talk about the negatives in their lives. But there’s an awful lot of people who, at Christmastime, you know, you get your Christmas cards and you get these letters with the Christmas cards. I absolutely hate that, I absolutely hate it. I mean, going back about three years ago, one particular school friend from when I was at school, … . And each year she writes and tells me what her two daughters are doing. And I know she wants to be proud of her children, but it makes me quite upset. And about three years ago, I wrote her a letter back, and I basically put it how it was. And she then got back in touch with me and said how sorry she was. And I said, ‘Don’t be sorry,’ I said, this is life and I just wanted you to know that not everybody’s life is as perfect, and for people not to judge. (Carer 3)

The caring role was further compounded by representations of drug and alcohol dependency in the British press “So, you know, you have to look at the bigger picture. And everybody, of course, believes everything that’s written in the newspapers. The newspapers do tend to sometimes manipulate the situation” (Carer 7). The unpaid carers that we spoke to were frustrated by the lack of understanding of drug and alcohol dependency that existed in society. One particular carer discussed how addiction can affect anyone and that people should not judge and assume the worst:

“You know, you should never, ever forget where you’ve come from because, at the spin of a coin, you could be in the gutter. And it’s alright walking past addicts, let’s put it as addicts” in the street and, “Oh God, look at them,” or, “They’re off their head,” or whatever. They’ve all got mums and dads, or had mums and dads, have grandma’s and grandads, maybe even brothers and sisters. And I feel it’s quite sad that people put them into a compartment, “Oh, they’re drug users so they’re no good. They’ll steal off you, they’ll do this, they’ll do that” (Carer 1)

The lack of understanding and misconceptions in society about drug and alcohol dependency was a constant challenge for the unpaid caregivers we spoke to. Feelings of blame, shame, and stigma negatively affected their caring role. Recognizing these complex and very specific challenges highlighted the need for support services to better understand the challenges unpaid carers face when accessing support and healthcare. Unpaid carers that we spoke to felt that support was limited and, therefore, they felt marginalized in their caring role.

### Marginalisation

The lack of support felt by unpaid carers heightened feelings of marginalization and intensified further challenges in their caring role, “Stigma, prejudice, isolation, anger and guilt are all the challenges that I face daily. No one seems to care that I have been thrust into this position. I talk to my Doctor and they just brush me aside” (Carer 6). These feelings of marginalization were often exacerbated by the feeling of little or no help from existing health and social care agencies. “There is no support for carers. I’m still waiting for carer needs assessment and I’m fighting the local authority with regard to legislation and my son’s rights to a care needs assessment” (Carer 2). This was further intensified during COVID-19 as one carer explained, “there is nothing to help me from the government agencies such as health or Social Care. When I asked if anything was available, I couldn’t even visit my Doctor because of COVID; I was prescribed antidepressants. I just needed to talk with someone, to pass the day away, [the project] helped me do this to some extent” (Carer 7). Being a parent career of a young person, the battles and struggles they experienced are voiced next:

You’re putting formal complaints to your local authority, and they ignore you, they fob^5^ you off … I’ve gone to the local authority ombudsman, and government ombudsman … but it’s taken me four years to get to that stage because I’ve had to learn the cycle how you’re dealt with, as a parent. That’s somebody who is used to dealing with professionals and that’s the hardest thing … so being a carer, you do not see it but it’s just that isolation … it’s like parents and carers of younger people and the lack of support, especially for under fourteens or under sixteens because you cannot possibly have a drug problem if you are under 16!. There is nowhere in the country that can support you, apart from private. (Carer 3)

To overcome marginalization and misconceptions, participants discussed that raising awareness about caring for someone with drug and alcohol dependence was imperative; “To be taken seriously, for the public to understand what it is to be a carer and what we give back to our society” (Carer 7). Unpaid caregivers often felt disregarded by medical professionals, and the persistence of blame further compounded this as a parent:

Professionals not being linked up, that’s always been my challenge and I challenge people, yes, I’m his mum, yes, he’s got drugs and alcohol problems, he’s adopted but I’m a person that actually isn’t going to put up with it, … it’s the blame and having to fight for everything … So, I’m that pain in the arse mother that keeps writing to the Chief Executive and things but then you get fobbed off as well. You have to play carefully because you are either a problematic mother, parent, or you are too emotional, and if you get emotional, you cannot do that because you have got to put your professional head on to deal with them. But as soon as you become emotional, you just get, neurotic mother or whatever and they just pigeonhole you. (Carer 2)

By further exploring and understanding the role of caring, unpaid carers were asked what would help overcome some of these challenges, recognizing their role as experts, and listening to their concerns and hopes for the future.

### Inclusion

Unpaid carers who we spoke to wanted a holistic family approach in the care, treatment, and recovery plan of the person that they were caring for so that they felt part of the solution and not excluded:

Well, you know, the minute our (sibling) turned 18, … we were not included. We did not know where she was at the time. You know, she lives with us. I think it was taken a bit far. They said, well, we can’t give you any information. We cannot tell you anything. We cannot involve you, you know, and we found that really challenging. (Carer 3)

Carers wanted to be involved at every stage of the recovery journey, recognizing them as experts and experienced. “What would be useful going forward is services joined up … this is what carers actually want and to highlight the importance of the carer as being involved.” (Carer 7). Being involved in the journey and treatment of care and by services working in partnership with one another was advocated by carers because not doing so exacerbated their already complex caring role. “The biggest challenge was about services not working with each other. We constantly had services saying … it was their problem, our problem, their problem, and our problem. And not involving us as carers” (Carer 4).

Unpaid carers felt that to be included in the journey of the person they were caring for, was only achievable through a joint approach for carer and cared for. This was often spoke about in terms of services working together in recognizing that alcohol and drug dependency should be treated alongside mental health. All the unpaid carers we spoke to had experienced caring for their loved ones and not being able to access mental health support in the treatment of care.

### Dual diagnosis (addiction and mental health)

In addition to caregivers advocating for a holistic family approach, caregivers also voiced that one of the most important in which ways their caring role would improve for them and the person they were caring for would be to also provide support for mental health alongside, not separate to, drug and alcohol dependence:

My feedback was that they were not specifically focused on mental health, but substance abuse, but not substance abuse with mental health, and they kept saying to us a number of times, ‘Your situation is different.’ Because we had a severe problem with mental health, and then substance abuse, and they can, you appreciate, come hand-in-hand. When dealing with substance abuse or alcohol, as we are, you know, you have to learn what to do. We did not know what to do. We knew about mental health; however, we did not know what to do with alcohol. So, I think people you can talk to is absolutely crucial, and people giving you confidence, of this is what you need to do. I think that was, which was crucial. We got that, and that’s what helped us in the end. (Carer 4)

This dual diagnosis of treating mental health alongside addiction was imperative for all the unpaid carers that we spoke to:

That’s been the hardest and actually dealing with my son’s mental health deteriorating and physical health and ADHD and PTSD and everything that comes with it and fighting for professionals to recognize the complexity and the needs. (Carer 2)

In co-occurring disorders, mental health issues and drug or alcohol addiction have unique symptoms that must be recognized and treated accordingly. This widespread recognition was reported by carers as one of the main challenges they experienced. A prominent area of concern was that unpaid carers felt that services were not recognizing that mental health is not a separate issue for drug and alcohol dependency. ‘And especially between the hospital mental health and the recovery aspects. They have got to work together, and their timing was really bad at times, it almost was that they were criticizing each other openly in front of us, so that is one thing’ (Carer 4). In summary, caregivers advocated for health care providers to work actively and closely together when considering dual diagnosis, treating addiction, and mental health together, to support caregivers of people with drug and alcohol dependence in a much more holistic way.

### Mental health and wellbeing: a caregivers perspective

Through talking with unpaid carers, issues with mental health and wellbeing were a key health concern for caregivers. The effect of caring for someone on family relationships and mental health was expressed by all unpaid carers; “I need time away to spend quality time as a family, which I don’t anymore, caring impacts on the family unit” (Carer 7). This was reiterated by another unpaid carer who expressed the following:

Basically, you put your child first, you give up your career and then you see your mental health and everything going down, so you can’t actually make a decision and that side of things but you’re struggling on. You go for help, well it’s a cup of tea and a chat and there’s no action, there’s no real support and the services aren’t out there. (Carer 2)

Unpaid carers wanted respite to enable them to continue in their caring role; “I have no time for myself. Need time away just for a few days just to sleep, just to recharge my batteries” (Carer 8). To help support and improve health and well-being, unpaid carers recognized the importance of looking after themselves:

It’s now my time to put myself and my [siblings] needs first, and to look after myself and my own health. Because I have to be honest, I comfort eat … which is massively unhealthy for me. So I have to put myself first. (Carer 1)

In recognizing how difficult it was for face-to-face communication and support from others during COVID-19, anonymous online platforms made a difference to caregivers’ lives because of connecting with others. These anonymous, safe online spaces that provided peer support as well as sharing and gathering information were enablers for caregivers to help with their caring role:

It is a fantastic place to be, everyone is kind and responsive, it is a little quiet sometimes but there is usually someone to chat with. I look after my [family member], who lives away now, so the pressure is not continuous like some … I still log on. It has made me feel connected somehow, especially when COVID was at its worst, it was good to feel this connection. (Carer 5)

In summary, the impact of caring for a loved one significantly affected unpaid carers’ mental health and well-being. Caregivers’ experiences, documented above, have identified a range of challenges specific to this cohort including stigma, shame and feelings of being judged by others, marginalization, a lack of understanding of drug and alcohol dependency from service providers, caring for complex needs including mental health conditions and the impact on family relationships. Caregivers also advocated for ways in which changes could be made to help improve their overall mental health and wellbeing and in overcoming barriers to accessing support from health service providers.

## Discussion

The caregivers the authors engaged with identified a range of challenges, suggestions, and concerns as documented above. Their experiences emphasized that there is still a culture of stigmatization around alcohol and drug addiction that can be negative and delimiting (NHS Addictions Provider Alliance NHS APA, 2021, p. 5). The caring role was further compounded by representations of drug and alcohol dependency in the British press where upon negative stereotypes around social health problem were often portrayed (UK Drug Policy Commission, 2010:57). In addition, unpaid carers discussed different types of stigma, the experience of being stigmatized by others (Stangl et al., 2019), social and/or public (NHS Addictions Provider Alliance NHS APA, 2021, p. 5), and felt marginalized from the anticipated bias expected from others similar to Kreek’s (2011) work. Caregivers experienced “prejudice and negative attitudes held by members of the public or society” (NHS Addictions Provider Alliance NHS APA, 2021, p. 5) which highlighted concerns about a lack of understanding around alcohol and drug addiction in the UK. Whilst the NHS is committed to reducing stigma and launched a public campaign in 2021 (see NHS Addictions Provider Alliance (NHS APA), 2021) it was evident from talking with unpaid caregivers still that stigmatized and discriminated against which “can make access to treatment very difficult” (NHS Addictions Provider Alliance NHS APA, 2021, p. 2). Williamson (2012), p. 1) argues that “adopting a medical approach will reduce the stigma that impedes the provision and acceptance of treatment”. However, Williamson (2012), p. 1) also reminds us that “the medical paradigm has existed for many years without significantly affecting the negative social attitudes that surround dependence” which this paper is in agreement with.

It has been recognized that families are important stakeholders in the treatment process (Miller & Wilbourne, 2002) and that there needs to be an “increased emphasis on the role of families and wider social networks in routine service provision” (Copello & Orford, 2002, p. 1361). Unpaid carers do not always identify with this term as usually, it was a family member that they were caring for, and this was part of being a “family”. Family members caring for a loved one with drug dependency have been referred to by Copello and Templeton ((2012), p. 1) as “the forgotten carers”. The needs of family members have been recognized in national policy and by others (Barnard, 2005; Copello et al., 2008) yet there are calls for greater recognition of substance misuse policies across the UK. Families have been identified as a valuable source of support for the treatment and recovery of the person they are caring for. However, apart from a few exceptions, “adult family members as a specific group are not yet clearly identified in policy and guidance” (Copello & Templeton, 2012, p. 1). This largely hidden group of forgotten carers is partly due to “concerns about stigma but also because their focus and that of drug treatment services has been first and foremost towards helping the person with the drug problem” (Copello & Templeton, 2012, p. 2). To put this in perspective the UKDPC estimated in 2008 in the UK, at the very least that “1.4 million adults were significantly affected by a relative’s drug use, including about 140,000 adult relatives of people in drug treatment” (UK Drug Policy Commission UKDPC, 2012, p. 131). Research also suggests that alcohol consumption and related harm increased during the first year of the COVID-19 pandemic (Cook et al., 2022; White et al., 2022) and did not return to pre-pandemic levels. Drug-related deaths have also been the highest since records began in 2020 (Office for National Statistics, 2024). It is therefore beneficial to deal with addiction in “a holistic way that takes into account the family context within which most people live” (Templeton et al., 2007, p. 137).

Mental health difficulties and substance misuse problems affect a significant proportion of the adult population and “approximately 50% of people with severe mental disorders are affected by substance misuse” (Robinson et al., 2023, p. 1). It is very common for people to experience problems with their mental health and drug/alcohol use at the same time, often referred to as a co-occurring disorder or dual diagnosis (Public Health England, 2017). It was first reported in 1990 that roughly 50% of individuals with severe mental disorders are affected by some form of substance abuse or dependence, and 37% of people who are dependent upon alcohol also have at least one serious mental illness (Regier et al., 1990, p. 2511). Public Health England (2017, p. 8) reported that mental health problems are experienced by the majority of drug (70%) and alcohol (86%) users in the UK. In co-occurring disorders, both mental health issues and drug or alcohol addiction have their own unique symptoms which must be recognized and treated accordingly. It is important to recognize that “substance dependence is not a failure of will or of strength of character but a medical disorder that could affect any human being. Dependence is a chronic and relapsing disorder, often co-occurring with other physical and mental conditions” (World Health Organisation, 2004, p. 247) which the same can be applied to alcohol dependence. Mental health difficulties and alcohol and drug dependency affect a significant proportion of the adult population,

As documented, the cost-of-living crisis in the aftermath of the COVID-19 pandemic has exaggerated inequalities for vulnerable communities, increasing the mental health crisis that was already prevalent in the UK. The overall number of people reporting mental health problems has increased in recent years, and mental health problems are a growing public health concern worldwide (World Health Organisation, 2022). In addition, there are significant challenges related to inequalities in health distribution and socio-demographics factors, where the role of the social environment should be central and as important as that played by individual factors (Orford, 2001 cited in Copello & Orford, 2002, p. 1361). Whilst alcohol misuse can be widespread and impact anyone, people of lower socioeconomic status show greater susceptibility to the harmful effects of alcohol and are more likely to die or suffer from a disease relating to their alcohol use (Public Health England, 2016, pp. 52–53). The Coalition government’s austerity programme, originating in 2010, has been attributed to the declining quality of care provided in recent years (Nuffield Trust, 2023). In analysing the harm suffered by millions of people in the UK, caused by austerity policies in response to the financial crisis of 2007–08, Cooper and Whyte (2017), p. 37) found a “significant, abrupt, and sustained increase in suicides following austerity-related events such as announcements of spending cuts”. Austerity policies have had damaging and harmful effects on people’s lives, which has only continued in the aftermath of COVID-19 and the continuing cost-of-living crisis. The unprecedented austerity measures that have since been witnessed have disproportionately impacted unpaid carers, and the challenges they experience can be viewed as direct social harm.

The complex and unique experiences documented by the caregivers in this paper, need to be contextualized and understood in terms of the harm that can be caused to an unpaid caregiver and the person being cared for. When speaking of “harms” they can be understood as the non-fulfilment of human needs (Doyal & Gough, 1984, 1991) which can be applied to unpaid carers. Pantazis and Pemberton (2009), p. 218) drew upon Doyal and Gough (1991) theory of human need and reported that humans require basic needs, including physical health (being free from physical ill health) and autonomy (sufficient mental health support and opportunities to act). Arguably, an individual is harmed through the non-fulfilment of their needs, which can be identified as needs at an individual level but can also be viewed through a series of structural and contextual needs (Pemberton, 2007).

Hillyard et al. (2004), p. 20) argue that the social harm approach includes the following categories of harm: physical, financial/economic, emotional/psychological, and cultural safety. Locating social policy debates around well-being and health is potentially of great utility to this social harm perspective because it provides an understanding of human needs and the conditions necessary for their fulfilment (Pemberton, 2007). This is an appropriate framework for exploring the harm experienced by unpaid carers which theoretically enables us to recognize that unpaid carers are being harmed as their needs are not being fulfilled or provided for.

Building upon this, the challenges that unpaid carers identified and experienced throughout this study can be categorized accordingly. The first is physical harm. Unpaid caregivers discussed feeling isolated and experiencing poor physical health because of the impact of their caring role. Second, financial and economic harm. The unpaid carers we spoke to were detrimentally impacted by the cost-of-living crisis and the cost of financially supporting their loved ones. Concurring with Carers (2024b), p. 5) “unpaid carers in the UK are providing care worth an unbelievable £184.3 billion a year … this means that unpaid carers are providing care equivalent to the budget of a second NHS in the UK”. Third, emotional/psychological harm was evident through unpaid caregivers discussing how their caring role was detrimental to their mental health. The factors that contributed to this were the discrimination they felt by professionals and the social and public stigma that had an emotional impact on their well-being. This has also been recognized by Pemberton ((2015), p. 30) where harms “resulting from enforced exclusion from social relationships/networks and from misrecognition are injurious in many ways”. Fourth, cultural safety is documented as harmful when access to cultural and intellectual information and resources is not provided. All of the unpaid carers that we spoke to voiced concern about not being supported and the battles and barriers they faced in accessing support from healthcare providers. Basic needs of “healthcare and intermediate needs of provision and access to appropriate preventative curative and palliative care” are all required to avoid harm (Pantazis & Pemberton, 2009, p. 218). It is only through recognizing that there are significant health inequalities across a range of vulnerable and marginalized communities, and by engaging with and listening to those who experience this that will help alleviate some harm that has already been caused.

## Conclusion

Some barriers to accessing support as a caregiver identified above, has been documented across other vulnerable communities that were part of this larger evaluation, for example, needing respite, not identifying as a “carer” and a lack of including unpaid carers in the health care journey of the cared for person (see Greenhow & Tickle, in press). In our discussion with unpaid carers from different communities, the importance of language around “carers” was a recurring theme as the majority of caregivers did not identify as a “carer”. The term “carer” therefore is a problematic and may be acting as a barrier to accessing support, particularly through promotional resources with “carer” in the title. Alternative inclusive language should be considered, such as “support for a loved one” instead so that people are able to identify so that people are able to identify when caring for someone.

Reflecting upon the unpaid carers experiences and drawing upon wider research in this paper, we can conclude that there is an urgent need to the complex needs of unpaid carers when caring for someone with drug and alcohol dependency as a distinct group of caregivers. Tailoring support to meet unique support needs is vital. Recognizing that an individualized and personalized approach is important when supporting carers, as not one size fits all, was a key message from this study, particularly when the cared for person is young. However, in addition to this, there are a range of specific challenges that our cohort of caregivers experienced, including stigma, shame and feelings of being judged by others, a lack of understanding of drug and alcohol dependency from service providers, caring for complex needs including mental health conditions and the impact on family relationships which this paper has intentionally documented upon.

There is a need to educate, raise awareness and destigmatize issues associated with drug and alcohol dependence to change societal perceptions so that unpaid carers receive the help and support they need. Caregivers reported a sense of stigma when caring for someone with alcohol and drug decency as they felt they did not receive the same level of empathy, understanding or compassion from friends and family and service providers. Raising awareness and understanding about the role of a caregiver is therefore two-fold; doing so to change societal perceptions with the public; and with professionals and service providers in understanding the complex and unique needs of the role of a caregiver. First, this paper advocates for further work to be conducted to engage with caregivers who are caring for someone with alcohol and drug dependency, whom because of societal perceptions around stigma are hard to reach. Second, to raise awareness to reduce stigma around alcohol and drug dependency and to improve the knowledge base about the specific needs of this cohort across health service providers and the public. Recognizing the complex challenges and needs when caring for someone with drug and alcohol dependency is imperative, so that the public, professionals and others can understand the role of a caregiver in understanding the impact this has on family and relationships with others. Focused information and training, closer partnership working and marketing are just some ways in which this can be achieved.

Recognizing the impact that COVID-19 had on an already vulnerable community of caregivers and the need to account for this in the future service provision is another key message. Bringing key services together and working closely with organizations in the locality for referrals to treatment and rehabilitation is key in order to make a difference long term. Projects sharing their findings and recommendations at networking events with partners, other support services, on social media channels and at training events were suggested to help improve internal practice of the organizations and also wider services.

Unpaid caregivers hoped that communication across all health services would improve and that they would be included in the recovery and treatment plan of the person they were caring for. Caregivers wanted to be involved at every stage of the recovery journey, recognizing them as experts and experienced, so that they can be part of the solution and be informed throughout the process. Knowledge and understanding about the experiences of people close to someone with alcohol and drug dependency is imperative in this regard. A joint and/or family approach for carer and cared for is needed to promote carer inclusion in the care of their loved ones.

Dual diagnosis, treating addiction alongside mental health issues, was voiced as a key concern for unpaid caregivers. Health services must work more actively and closely together when considering dual diagnosis, treating addiction and mental health together to support carers of people with drug and alcohol dependence in a more holistic way. This will not only improve the journey and treatment of the person being cared for but will simultaneously improve the mental health and well-being of the unpaid carer, who, through their narratives above, have documented the detrimental impact that caring can have. Projects involved in the evaluation acknowledged this and were committed to embedding these key message and concerns. Raising awareness, services working together through wider service delivery and provision were discussed as ways forward.

The detrimental impact of caring on well-being, as noted above, resonates with findings published year-on-year by Carers UK (2023), p. 05), who have evidenced that mental health and well-being are being damaged as a result of caring—some of which is preventable with the right interventions, information, advice, and support from the NHS, social care, and the wider voluntary sector. Respite, meeting with others, and peer support were all potential coping mechanisms and what caregivers wished for to help improve their own health (mental and physical) in the future. Ensuring that support groups continued and funding, to provide these support services, were voiced as being vital to improve wellbeing.

The projects evaluated were all committed to continuing closer partnership working, recognizing that there needed to be a change in culture and practice to remove barriers for caregivers who were accessing support to health service provision. However, it was recognized that sustainability of these outcomes was dependent upon a number of factors which included funding and resources, networking and partnership working where buy-in from wider services was needed and further understanding was needed about the complex caring role. Therefore, in order for recommendations and interventions to continue and be sustained, there is a need for support from a range of partners. It is only through understanding the complex and unique set of challenges faced by unpaid carers caring for someone with alcohol and drug dependency, that these issues can be addressed. Recognizing that some common issues raised here in this paper require further consideration in the future is detrimental to alleviating a range of social harms that caregivers experience on a daily basis.

## Data Availability

Due to the nature of the research and participant privacy considerations, supporting data were not available.

## Notes

1. Carers from The Equality Act (2010) protected groups were identified by NHS England and NHS Improvement [NHSE/I] as vulnerable: age; disability; gender reassignment; marriage and civil partnership; pregnancy and maternity; race; religion or belief; sex; sexual orientation; alcohol and substance addicts; armed forces families; migrants such as refugees and asylum seekers.

2. Unpaid carers who participated from this project will be referred to as 1–4 throughout this paper.

3. Unpaid carers who participated from this project will be referred to as 5–6 throughout this paper.

4. Unpaid carers who participated from this project will be referred to as 7–8 throughout this paper.

5. To cheat, deceive, delude. To fob a person of or out of something. Colloquial (Oxford Dictionary 2025).

## Appendix 1. Research aims of Mind The Gap

Research question:

What are the barriers to accessing support services for unpaid carers from vulnerable communities?

Research Aims:

1. To understand the current state support services for carers in England.
2. To understand the value of support services for carers from diverse communities.
3. To understand the experiences of carers when accessing support services.
4. To evaluate what works when engaging and supporting carers from diverse communities.

## Appendix 2. Semi-structured interview schedule for all participants

- Please could you tell us about your experience of being involved in Mind The Gap?
- What is most important to you to help to maintain your own health and wellbeing?
- Has this been identified/addressed/improved through Mind The Gap?
- What barriers/challenges do you face when caring for someone?
- Have these been overcome or identified through Mind the Gap?
- Would you recommend the outcomes from Mind The Gap for other caregivers?
- What matters most to you in the future?
